# Dandy-Walker Malformation Presenting with Psychological Manifestations

**DOI:** 10.1155/2016/9104306

**Published:** 2016-07-14

**Authors:** Yasodha Maheshi Rohanachandra, Dulangi Maneksha Amerasinghe Dahanayake, Swarna Wijetunge

**Affiliations:** ^1^Department of Psychiatry, Faculty of Medical Sciences, University of Sri Jayewardenepura, Gangodawila, 10250 Nugegoda, Sri Lanka; ^2^Child and Adolescent Mental Health Services, Lady Ridgeway Hospital for Children, 800 Colombo, Sri Lanka

## Abstract

Dandy-Walker malformation, which is a congenital malformation of the cerebellum, is documented in literature to be associated with psychotic symptoms, obsessive compulsive symptoms, mood symptoms, hyperactivity, and impulsive behavior. The pathogenesis of psychiatric symptoms in Dandy-Walker malformation is thought to be due to disruption of the corticocerebellar tracts, resulting in what is known as cerebellar cognitive affective syndrome. We present a case of Dandy-Walker malformation presenting with psychiatric symptoms. This case highlights the necessity to be aware of psychiatric manifestations of cerebellar disease as it has an impact on the diagnosis and treatment.

## 1. Introduction

Dandy-Walker malformation (DWM) is a congenital malformation of the cerebellum characterized by hypoplasia of the cerebellar vermis, cystic dilatation of the fourth ventricle, and enlarged posterior fossa. Dandy-Walker variant is a condition with variable hypoplasia of the cerebellar vermis with or without enlargement of the cisterna magna, communication between the fourth ventricle and the arachnoid space, and no hydrocephalus [1]. It is a milder form of Dandy-Walker syndrome, with less severe radiological abnormalities including the absence of hydrocephalus, less neurological symptoms, and better outcome [2]. Several case reports have shown Dandy-Walker variant malformation to be associated with psychotic symptoms, obsessive compulsive symptoms, mood symptoms, hyperactivity, and impulsive behavior [1, 3–8]. We report a 13-year-old girl who presented with psychotic symptoms, obsessive compulsive symptoms, personality changes, and mood changes, who showed poor response to medication and was later found to have Dandy-Walker variant malformation.

## 2. Case Presentation

A 13-year-old girl presented with deteriorating school performance for 2 years with repetitive impulses to touch others inappropriately and believing that there is a devil living inside her body for a duration of 6 months.

She has had normal developmental milestones and average school performance for 2 years back. Over the past 2 years, she had inattention, lack of interest in school work, and deteriorating school performance. During the 6 months prior to presentation, she had poor sleep, had become irritable, and had developed grandiose delusions, claiming that she had attained nirvana. She also had bizarre delusions with somatic hallucinations, believing that a devil was residing inside her and feeling this devil's movements. She had episodes of aggression and attributed these to the devil, which was consistent with a delusion of control. There were no mood symptoms. Over this period, she had obsessional impulses to perform certain acts of sexual nature. A diagnosis of schizophrenia with obsessive compulsive symptoms was made and she was commenced on risperidone 2 mg at night and escitalopram 20 mg in the morning.

Over time, she showed poor response to medication and started refusing to go to school as she believed her classmates were talking about her. She started acting out on her obsessional impulses and tried to touch her classmates inappropriately. She displayed odd behaviors such as putting match sticks in her dog's mouth and sleeping on the roof.

On mental state examination, she was disinhibited evident by her behavior during the interview where she made inappropriate sexual advances towards the interviewer. Her speech lacked prosody. Her mood was flat. She had persecutory delusions and ideas of reference regarding her classmates. She had obsessional impulses to touch others inappropriately, which she resisted minimally. There were no perceptual abnormalities. Her verbal fluency was reduced. Other frontal lobe functions measured by categorical fluency, proverb interpretation, Luria 3-step test, go/no-go test, and written sequencing were normal. Parietal and temporal lobe functions measured by left-right orientation, clock drawing test, and drawing reproduction and memory measured by recall of 5-item address were normal. Nonverbal IQ measured by TONI-3 (Tests of Nonverbal Intelligence-3) was normal. Physical examination revealed bradykinesia, but no rigidity, hyperreflexia, or reduced muscle power. There were no abnormal movements, cerebellar signs, or sensory deficits. Examinations of the cardiovascular, respiratory systems, and the abdomen were unremarkable.

**Figure 1:**
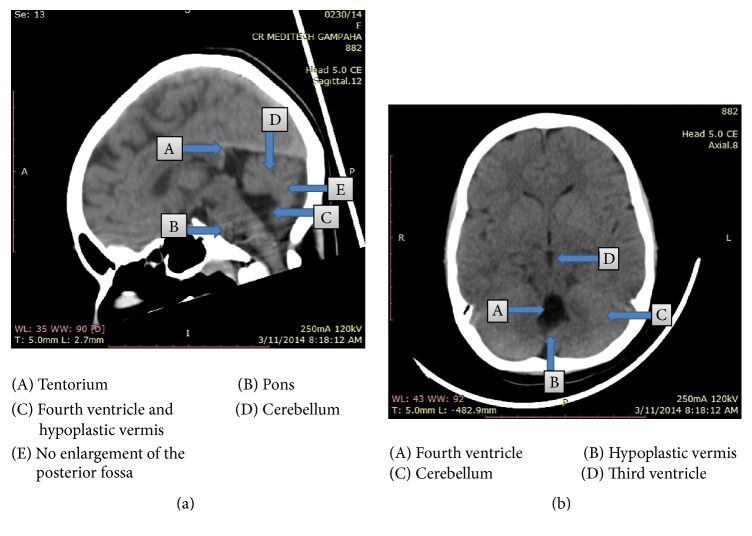
CT images of the patient, showing Dandy-Walker malformation.

Her investigations revealed the following. Full blood count, blood picture, and erythrocyte sedimentation rate done with a view to detect any underlying inflammatory conditions including autoimmune disorders were normal. Tests for thyroid function were done to rule out psychiatric manifestations arising due to thyroid dysfunctions. Both free thyroxine and thyroid stimulating hormone were normal (free thyroxine 1.12 ng/dL and thyroid stimulating hormone 3.87 IU/L). Electroencephalography recorded in the alert state was normal for the age. An eye referral excluded the presence of Kayser-Fleischer rings. Cerebrospinal fluid lactate done to exclude mitochondrial diseases was normal. Computerized tomography brain showed an asymmetrically dilated 4th ventricle, with a cerebrospinal fluid filled cleft connected to right sigmoid sinus, bisecting cerebellar hemispheres which were poorly formed (Figure 1). Appearance was consistent with a Dandy-Walker syndrome variant.

Risperidone was increased to 2 mg twice daily and escitalopram continued at the same dose. Over 6 months of treatment, her psychotic symptoms, disinhibition, and obsessions showed partial response and she continued to have odd behaviors with deterioration of school performance. The option of changing the antipsychotic to olanzapine or quetiapine was discussed with the patient and her family. However, they preferred continuing the current medication due to the higher incident of side effects of weight gain and sedation associated with these medications. In addition, commencing a newer antipsychotic such as aripiprazole, which is not available in the government sector in Sri Lanka, was considered, but family was unable to afford it. Neurology opinion was sought and it was decided that no active surgical interventions were indicated at this stage.

## 3. Discussion

The first case of schizophrenia-like-psychosis in Dandy-Walker variant was reported by Turner et al. in 2001 [9]. Since then, several case reports have described psychosis in all four subtypes of DWM [3–5]. Psychosis associated with DWM is described to be characterized by juvenile or young adult onset, atypical psychotic symptoms, high frequency of family history of psychosis, high prevalence of cognitive deficit, and refractoriness to treatment. Of the above features, the patient we described had a juvenile onset, cognitive deficits, and responded poorly to treatment.

Schizophrenia with obsessive compulsive symptoms is also recognized in DWM. Numerous case reports have linked DWM to schizophrenia with obsessive compulsive symptoms [6, 10, 11], similar to the case presented above. However, as it has been suggested that since obsessive compulsive disorder is a common occurrence in schizophrenia, OCD symptoms may occur coincidentally and need not necessarily be viewed as arising from the associated cerebellar malformation [11].

Furthermore, literature survey revealed a report of a patient with recurrent depressive disorder with impulse control difficulty in the presence of DWM, with impulsive behavior showing partial response to treatment with sodium valproate and quetiapine [1]. Impulsive behavior was also noted in the patient we described, with partial response to risperidone. Several reports showed an association between Dandy-Walker variant and bipolar disorder, which was associated with treatment resistance [7, 12, 13].

The function of the cerebellum in coordination and integration of motor activity is well established. In addition, its role in higher order functioning is increasingly being recognized. Cerebellar cognitive affective syndrome (CCAS) is used to describe the psychological dysfunctions postulated to arise from disrupted corticocerebellar circuits [14]. This may arise due to either congenital or acquired lesions of the cerebellum [14].

CCAS has been described as having symptoms in cognitive and psychiatric domains [14]. The cognitive symptoms include executive dysfunction, impaired spatial cognition, personality change, and linguistic difficulties [15]. Psychiatric features reported are depression, aggression, psychotic features, and obsessive compulsive symptoms [1, 15].

A previous case report has described a patient with Dandy-Walker variant presenting with symptoms of CCAS [16]. This patient had presented with learning disabilities, impulsiveness, attention deficits, impairment in working memory, executive dysfunction in planning and problem solving along with depressed mood, blunted affect, and psychomotor tension. As cerebellar dysfunction resulting in CCAS could explain all the clinical manifestations, it was suggested that the symptoms be viewed as a part of CCAS integral to DWM [16].

A number of the features that have been described in CCAS, including decreased verbal fluency, personality change with flattening of affect, impulsivity, obsessions, inappropriate behavior, disinhibition, and psychotic symptoms were found in the patient we described. Although schizophrenia with obsessive compulsive symptoms could be diagnosed in this patient, it does not fully explain the clinical picture. Hence, taking the above evidence into consideration, it appears possible to postulate that Dandy-Walker variant malformation presented as CCAS in this patient.

This report highlights the need for a complete evaluation, including imaging studies, in a child or adolescent presenting with first-episode psychosis. It also highlights the necessity to be aware of psychiatric manifestations of cerebellar disease, as it has an impact on the diagnosis and treatment and also has prognostic implications for the affected individuals.

Despite several reports in literature, the optimal pharmacological management strategies for those patients not requiring surgery are yet to be elucidated. A single study has examined the efficacy of aripiprazole in treatment of CCAS in a patient following resection of a posterior fossa tumor [17], which showed a therapeutic benefit of aripiprazole in treatment of mental state changes associated with CCAS. Further research is required to establish the best pharmacological options.

The course of these psychiatric manifestations has not been recognized. Further research is needed in these areas to optimize the quality of care offered to these patients.

## References

[1] Kim J. H., Kim T. H., Choi Y. C., Chung S.-C., Moon S. W. (2013). Impulsive behavior and recurrent major depression associated with Dandy-Walker variant. *Psychiatry Investigation*.

[2] Sasaki-Adams D., Elbabaa S. K., Jewells V., Carter L., Campbell J. W., Ritter A. M. (2008). The Dandy-Walker variant: a case series of 24 pediatric patients and evaluation of associated anomalies, incidence of hydrocephalus, and developmental outcomes. *Journal of Neurosurgery: Pediatrics*.

[3] Gan Z., Diao F., Han Z. (2012). Psychosis and dandy-walker complex: report of four cases. *General Hospital Psychiatry*.

[4] Buonaguro E. F., Cimmarosa S., De Bartolomeis A. (2014). Dandy-Walker Syndrome with psychotic symptoms: a case report. *Rivista di Psichiatria*.

[5] Ryan M., Grenier E., Castro A., Nemeroff C. B. (2012). New-onset psychosis associated with Dandy-Walker variant in an adolescent female patient. *The Journal of Neuropsychiatry and Clinical Neurosciences*.

[6] Papazisis G., Mastrogianni A., Karastergiou A. (2007). Early-onset schizophrenia and obsessive-compulsive disorder in a young man with Dandy-Walker variant. *Schizophrenia Research*.

[7] Aimua F., Dunn N. R., Swift R. G. (2012). Dandy walker variant with treatment-resistant bipolar disorder. *Journal of Neuropsychiatry and Clinical Neurosciences*.

[8] Sharma S., Vaish S., Kamal S., Usman N. (2014). Rare coexistence of Dandy-Walker malformation, down syndrome and hypothyroidism in a hyperactive child—a case report. *Journal of Indian Association for Child and Adolescent Mental Health*.

[9] Turner S. J., Poole R., Nicholson M. R., Ghadiali E. J. (2001). Schizophrenia-like psychosis and Dandy-Walker variant. *Schizophrenia Research*.

[10] Papazisis G., Mastrogianni A., Karastergiou A. (2007). Early-onset schizophrenia and obsessive–compulsive disorder in a young man with Dandy-Walker variant. *Schizophrenia Research*.

[11] Kani A. S., Poyraz C. A., İnce E., Duran A. (2015). comorbid schizophrenia and obsessive compulsive disorder associated with mega cisterna magna: a case report. *Yeni Symposium*.

[12] Babaki M. E. S., Estilaee F. (2015). Bipolar disorder in a young girl with Dandy-Walker syndrome. *Iranian Journal of Psychiatry*.

[13] Can S. S., Uğurlu G. K., Çakmak S. (2014). Dandy Walker variant and Bipolar I disorder with graphomania. *Psychiatry Investigation*.

[14] Schmahmann J. D., Sherman J. C. (1998). The cerebellar cognitive affective syndrome. *Brain*.

[15] Wolf U., Rapoport M. J., Schweizer T. A. (2009). Evaluating the affective component of the cerebellar cognitive affective syndrome. *Journal of Neuropsychiatry and Clinical Neurosciences*.

[16] Graf H., Franke B., Abler B. (2013). Cerebellar cognitive affective syndrome in Dandy-Walker variant disorder. *Journal of Neuropsychiatry and Clinical Neurosciences*.

[17] Yap J. L., Wachtel L. E., Ahn E. S., Sanz J. H., Slomine B. S., Pidcock F. S. (2012). Treatment of cerebellar cognitive affective syndrome with aripiprazole. *Journal of Pediatric Rehabilitation Medicine*.

